# Metabolic effects of reduced growth hormone action in fatty liver disease

**DOI:** 10.1007/s12072-018-9893-7

**Published:** 2018-09-11

**Authors:** Kerstin Rufinatscha, Claudia Ress, Sabrina Folie, Simone Haas, Karin Salzmann, Patrizia Moser, Jochen Dobner, Guenter Weiss, Paula Iruzubieta, María Teresa Arias-Loste, Javier Crespo, Herbert Tilg, Susanne Kaser

**Affiliations:** 1 Department of Internal Medicine I, Medical University Innsbruck, Anichstrasse 35, 6020 Innsbruck, Austria; 2 Christian Doppler Laboratory for Metabolic Crosstalk, Department of Internal Medicine I, Medical University Innsbruck, Innsbruck, Austria; 3 Institute of Pathology, Medical University Innsbruck, Innsbruck, Austria; 4 Department of Internal Medicine II, Medical University Innsbruck, Innsbruck, Austria; 5 Gastroenterology and Hepatology Unit, Hospital Universitario Marqués de Valdecilla, Instituto de Investigación Valdecilla (IDIVAL), Valdecilla, Spain; 6 Facultad de Medicina, Universidad de Cantabria, Santander, Spain; 7 Department of Gastroenterology, Hepatology and Infectious Diseases, University Hospital, Heinrich-Heine University, Düsseldorf, Germany; 8 Institute of Physical Biology, University of Düsseldorf, Düsseldorf, Germany

**Keywords:** NASH, obesity, Growth hormone receptor, Insulin signaling

## Abstract

**Background:**

Adult growth hormone (GH) deficiency is associated with fatty liver disease and shows several features of the metabolic syndrome. Vice versa obesity is characterized as a state of low GH function. Here, we aimed to define the role of hepatic GH signaling and its metabolic consequences in non-alcoholic fatty liver disease.

**Methods:**

In humans, GHR and IGF-1 levels were determined in liver samples of 29 obese patients with non-alcoholic steatohepatitis (NASH) or simple steatosis. Cellular effects of GH on insulin signaling were investigated in GH receptor (GHR) knockdown HepG2 cells.

**Results:**

Hepatic IGF-1 expression levels reflecting GH action were significantly lower and fasting glucose concentrations higher in patients with NASH than in patients with simple steatosis. GHR knockdown in hepatocytes resulted in a scenario of high glucose output displayed by reduced glycogen content, increased gluconeogenesis and diminished insulin signaling.

**Conclusions:**

Our data suggest that GH signaling in the liver is diminished in patients with NASH and associated with deteriorated hepatic insulin sensitivity and metabolic activity. Reduced hepatic GH action might contribute to insulin resistance in obese patients with NASH.

## Introduction

Non-alcoholic fatty liver disease (NAFLD) is strongly associated with insulin resistance characterized by reduced whole-body, adipose tissue and hepatic insulin sensitivity [1].

Adult growth hormone deficiency (AGHD) is characterized by increased visceral obesity, dyslipidemia, premature atherosclerosis and increased mortality [2]. Fatty liver disease is frequently found in patients with AGHD [3]. Accordingly, in some studies, GH and IGF-1 concentrations were decreased in patients with NAFLD [4, 5]. Clinically, GH replacement could dramatically ameliorate steatosis, inflammation and fibrosis in a patient with AGHD and NASH [6].

In mice, hepatocyte-specific deletion of GHR was associated with a fourfold increase in circulating GH levels and a strong suppression of IGF-1 levels [7]. Phenotypically, these mice are characterized by insulin resistance, glucose intolerance, hepatic steatosis and increased levels of free fatty acids. Cordoba-Chacon and colleagues reported tissue-specific direct effects of GH on glucose metabolism: GH levels were associated with insulin-mediated suppression of hepatic gluconeogenesis but negatively correlated with insulin-driven glucose uptake in the skeletal muscle in a mouse model of adult onset of isolated GH deficiency [8]. Liu and colleagues recently suggested from liver-specific GHR-knockout mice with restored IGF-1 expression that GH exerts direct effects on hepatic lipid metabolism [9].

Here, we investigated hepatic growth hormone metabolism in patients with NASH and also studied its metabolic consequence in a cell culture model of GHR knockdown.

## Materials and methods

### Cell culture experiments and siRNA transfection

HepG2 cells (ATCC, Manassas, Virginia, USA) cultured in a RPMI 1640 medium (Lonza, Basel, Switzerland) were transfected with predesigned small interfering RNA (siRNA) targeting human (h) GHR or non-silencing control siRNA (Qiagen, Hilden, Germany). Transfected cells were maintained in transfection medium containing up to 100 nM siRNA, 100 µl RPMI 1640 with 5% glutamine and 18 µl HiPerfect (Qiagen) for 72 h. The silencing effect of GHR was quantified by Western blotting analysis. All transfection experiments were performed in duplicates.

### Western blot analysis

Ten µg of total cell protein [10] or marker was run on a 4–15% gradient sodium dodecyl sulfate–polyacrylamide gel (BioRad, Hercules, USA) and blotted onto a nitrocellulose membrane (Hybond-P, Amersham-Pharmacia, Austria). Human GHR, human proteinkinase B (hAKT) and human phospho-proteinkinase B (hpAKT) were detected using commercially available monoclonal antibodies (Abcam, Cambridge, New England; Cell Signalling Technologies, Cambridge, New England). The membranes were incubated with, rabbit anti-hAKT, rabbit anti-hpAKT (Ser473) or rabbit anti-hpAKT (Thr308) (CellSignalling) antibodies at 4 °C shaking overnight. Target proteins were visualized by incubation with horseradish peroxidase conjugated (HRP) secondary antibody allowing (Jackson ImmunoResearch, West Baltimore Pike, USA; GE Healthcare, Vienna, Austria) and normalized to β actin protein abundance (Sigma-Aldrich, St. Lois, US).

### M-RNA expression analysis

Total RNA was extracted from frozen liver samples by the acid guanidinium phenol chloroform method which had been collected by Tru-cut from each subject before. Isolated total RNA from cell lysates or liver samples was transcribed into cDNA (Qiagen) and then quantified using SYBR Green-based real time PCR or the TaqMan real-time PCR method. Commercially available predesigned oligonucleotides for IGF-1 (Biorad, Hercules, USA) and GAPDH (TaqMan by Life Technologies, USA) were used. For designing primer and probes, the Primer Express Software (Perkin-Elmer Applied Biosystems, Warrington, US), National Center for Biotechnology Information (NCBI, Rockville Pike, US) and GenScript (GenScript, New Jersey, US) were used. Designed probes and primers (fatty acid synthase (FAS), glucose-6-phosphatase (G6Pase), pyruvate carboxylase (PC), phosphoenolpyruvate carboxykinase (PCK1), diacylglycerol O-acetyltransferase 2 (DGAT2), peroxisome proliferator activated receptor γ and α (PPARγ, PPARα), stearoyl-CoA desaturase (SCD-1), carnitine palmitoyl transferase 1 (CPT-1) and sterol regulatory element binding protein 1c (SREBP-1c)) were purchased from Microsynth (Microsynth, Baglach, Switzerland). Sequences are available upon request. TaqMan real time PCR was performed using the TaqMan Universal Polymerase Mastermix (Applied Biosystems, Foster City, California, USA) and labeled probes (5′end: 6-carboxyfluorescein (FAM), 3′end: 6- carboxy- tetramethyl- rhodamine (TAMRA)). SYBR-green dye real time PCR was performed using SYBR Green PCR Kit (Qiagen). Conditions of amplification were 10 min at 95 °C followed by 40 cycles for 30 s at 95 °C and 1 min at 60 °C. Results were expressed as target gene/GAPDH cDNA ratio.

### Glycogen content

All experiments were performed in GHR siRNA, non-silencing siRNA transfected cells and untreated control cells in duplicates. After incubation of cells with or without insulin [100 nmol/l] for 3 h at 37 °C, glycogen content was determined by a method described by Decker et al. [11]. After washing with ice-cold PBS, cells were collected in 0.6 mol/l HClO_4_, sonicated on ice water and neutralized in 1 mol/l KHCO_3_. Aliquots of the homogenate were incubated with 10 g/l amyloglucosidase in 0.2 mol/l acetate buffers for 2 h at 40 °C. By adding chilled 2 mol/l HClO_4_ , the reaction was stopped and the mixture centrifuged at 14000 g at 4 °C for 10 min. Glucose concentrations were determined using a Cobas Mira analyser (Roche). Glycogen content was expressed as nmol glucose/mg protein. All experiments were performed with and without addition of IGF-1 (Sigma, St. Louis, USA) at a concentration of 20, 100 or 200 µg/ml for 2 or 24 h when indicated.

### Study population

Previously, 59 obese patients aged between 19 and 60 years with either histologically proven NASH or simple steatosis undergoing bariatric surgery were evaluated preoperatively and liver-biopsied intraoperatively [12]. In our study, only female subjects with all data and additional tissue specimen available were included for further evaluation. Finally, 14 women with simple steatosis and 15 females with NASH matched for BMI and age were included in our study. NASH was diagnosed as a clinical and pathological entity characterized by the presence of steatosis and lobular and/or portal inflammation with or without fibrosis [13]. None of the patients had signs of hepatic decompensation, heart failure, organic renal failure, pituitary or adrenal disorders, autoimmune disorders or cancer. Two patients with simple steatosis and 5 patients with NASH were diagnosed with type 2 diabetes. Fasting plasma values of liver function tests, creatinine, glucose and lipids were determined with standard clinical automated analyzers.

### Statistical analysis

All statistical analyses were performed with the statistical analysis software package SPSS version 17.0 (SPSS Inc., Chicago, USA). Descriptive data are expressed as mean ± standard deviation (SD). ANOVA analysis with Bonferroni correction or unpaired two-tailed Students *t* test for parametric data was performed to test for differences in cell culture experiments. Normality of data was assessed by the Kolmogorov–Smirnov test. Non-normal data were analyzed using the Mann–Whitney test or the Kruskal–Wallis test. For all calculations, statistical significance was inferred at a two-tailed p value of less than 0.05.

## Results

### Human data

Hepatic GHR and IGF-1 mRNA expression levels were determined in liver biopsies of 29 obese women undergoing bariatric surgery. Age and BMI were similar in 15 patients with NASH and 14 patients with simple steatosis (Table 1). Two patients with simple steatosis and 5 patients with NASH were diagnosed with type 2 diabetes. When patients with diabetes were excluded from this analysis, fasting glucose levels were similar in non-diabetic patients with NASH and simple steatosis (106.40 ± 16.04 vs 93.83 ± 14.96, *p* = 0.059). G6Pase/GAPDH cDNA levels tended to be higher in patients with NASH than in patients with simple steatosis without reaching statistical significance (1.42 ± 0.96 vs. 0.97 ± 0.58, *p* = 0.18).

**Table 1:** Characteristics of patients with NASH (*n* = 15) and subjects with simple steatosis (*n* = 14)

	NASH group	Simple steatosis group	*p* value
Age	45.1 ± 8.9	37.8 ± 11.8	0.07
BMI, kg/m^2^	45.4 ± 5.4	47.6 ± 10.6	0.48
Fasting glucose, mg/dl	139.5 ± 68.6	96.0 ± 17.2	0.03
Fasting total cholesterol, mg/dl	203.2 ± 39.4	194.8 ± 39.3	0.57
Fasting triglycerides, mg/dl	174.9 ± 164.7	102.5 ± 44.6	0.19
AST, U/l	22.7 ± 10.1	23.8 ± 7.0	0.75
ALT, U/l	31.5 ± 22.0	29.5 ± 17.9	0.80
γGT, U/l	42.8 ± 50.2	42.9 ± 41.7	0.14
Serum creatinine, mg/dl	0.90 ± 0.2	0.8 ± 0.2	0.19

**Fig. 1:**
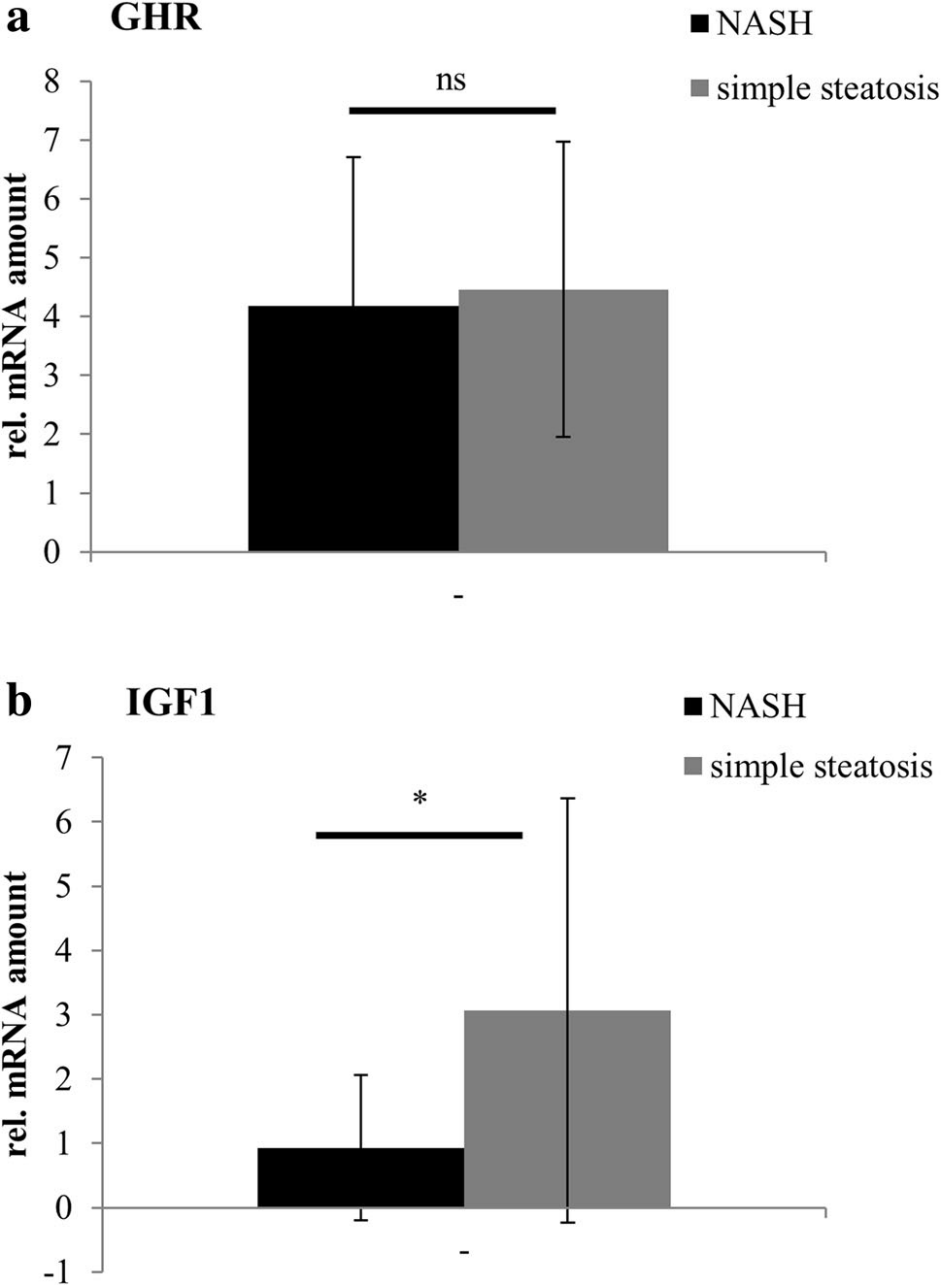
Hepatic GHR and IGF-1 expression in patients with NASH or simple steatosis. M-RNA expression levels of GHR (**a**) and IGF-1 (**b**) in liver samples of 15 obese patients with NASH and 14 obese patients with simple steatosis are shown. Target gene mRNA expression was normalized to GAPDH mRNA expression levels

GHR mRNA levels were comparable in patients with NASH and simple steatosis, while IGF-1 mRNA was significantly reduced in patients with NASH when compared to patients with simple steatosis (Fig. 1a, b).

When subjects were divided into 3 groups with respect to grade of steatosis, hepatic IGF-1 expression levels significantly decreased with advanced grade of steatosis (IGF-1/GAPDH cDNA ratio: steatosis grade 1 (*n* = 19): 2.72 ± 3.02; steatosis grade 2 (*n* = 5): 1.07 ± 1.52; steatosis grade 3 (*n* = 5): 0.56 ± 0.28, *p* = 0.01). Although GHR expression levels were highest in patients with steatosis grade 1, there was no significant difference in patients with steatosis grade 2 and 3 (GHR/GAPDH cDNA ratio: steatosis grade 1: 4.65 ± 2.22; steatosis grade 2: 3.18 ± 1.92; steatosis grade 3: 4.15 ± 3.86, *p* = 0.19).

When 15 patients with NASH were analyzed according to the grade of inflammation, IGF-1 expression decreased with advanced grade of inflammation; however, statistical significance was missed due to low sample size (IGF-1/GAPDH cDNA ratio: grade of inflammation 1 (*n* = 2): 2.07 ± 2.40; grade of inflammation 2 (*n* = 9): 0.91 ± 1.01, grade of inflammation 3 (*n* = 4): 0.41 ± 0.26; *p* = 0.25).

GHR expression levels tended to be lower in patients with highest grade of inflammation also (GHR/GAPDH cDNA ratio: 2.98 ± 1.88 vs 4.64 ± 2.95 (grade of inflammation 2) vs 4.52 ± 1.09 (grade of inflammation 1), *p* = 0.58).

When 7 patients with overt diabetes (5 with NASH and 2 with simple steatosis) were excluded from statistical analysis, similar results were observed. GHR expression levels were comparable in patients with simple steatosis and NASH (GHR/GAPDH cDNA ratio: 3.94 ± 1.82 in simple steatosis vs 3.53 ± 1.42 in NASH). IGF-1 mRNA expression levels were significantly lower in livers of patients with NASH (IGF-1/GAPDH cDNA ratio: 1.07 ± 1.34 in NASH vs 2.89 ± 3.55 in simple steatosis, *p* = 0.04).

### In vitro data

**Fig. 2:**
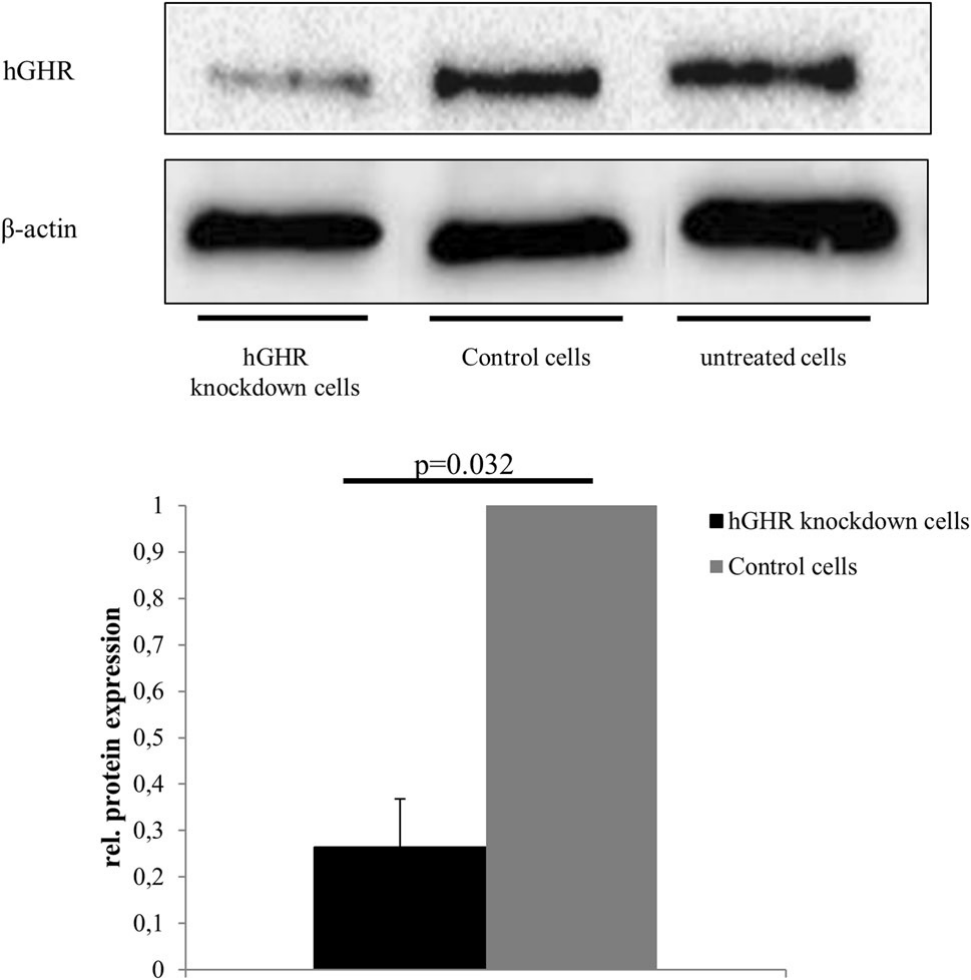
GHR knockdown efficacy in hGHR siRNA-transfected HepG2 cells

Cellular effects of GH were studied in a cell culture model of GHR knockdown. Treatment of HepG2 cells with 100 nmol GHR siRNA for 72 h resulted in significantly reduced GHR expression by 75 ± 5% (*n* = 7, *p* = 0.03) as determined by Western blot analysis when compared to HepG2 cells treated with non-silencing siRNA (Fig. 2).

**Fig. 3:**
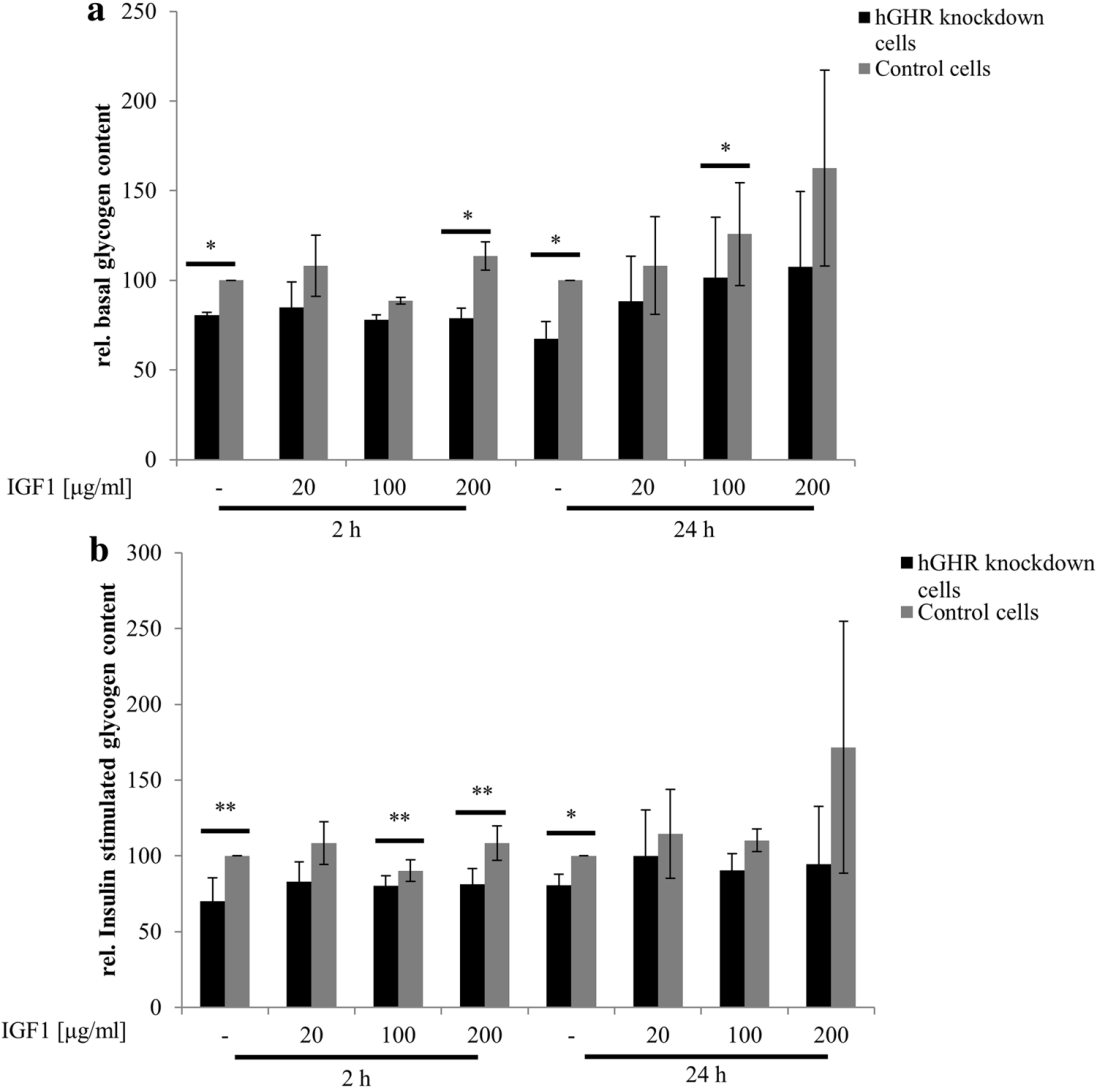
Glycogen content in GHR-expression-knock down cells. Basal (**a**) and insulin-stimulated (**b**) glycogen content was determined in GHR-knockdown cells and control cells transfected with non-silencing siRNA. To investigate whether IGF-1 compensates for reduced cellular GH action, cells were preincubated with IGF-1 at indicated concentrations for 2 or 24 h

Insulin sensitivity was estimated by basal and insulin-stimulated glycogen content. Both basal and insulin- stimulated glycogen contents were significantly lower in GHR-deficient HepG2 cells when compared to HepG2 cells transfected with non-silencing siRNA. To investigate whether effects are dependent on IGF-1 action, cells were preincubated with IGF-1 at indicated concentrations for 2 or 24 h. Short- and long-term preincubation of IGF-1 partly, but not completely, restored glycogen contents at all indicated concentrations as shown in Fig. 3.

**Fig. 4:**
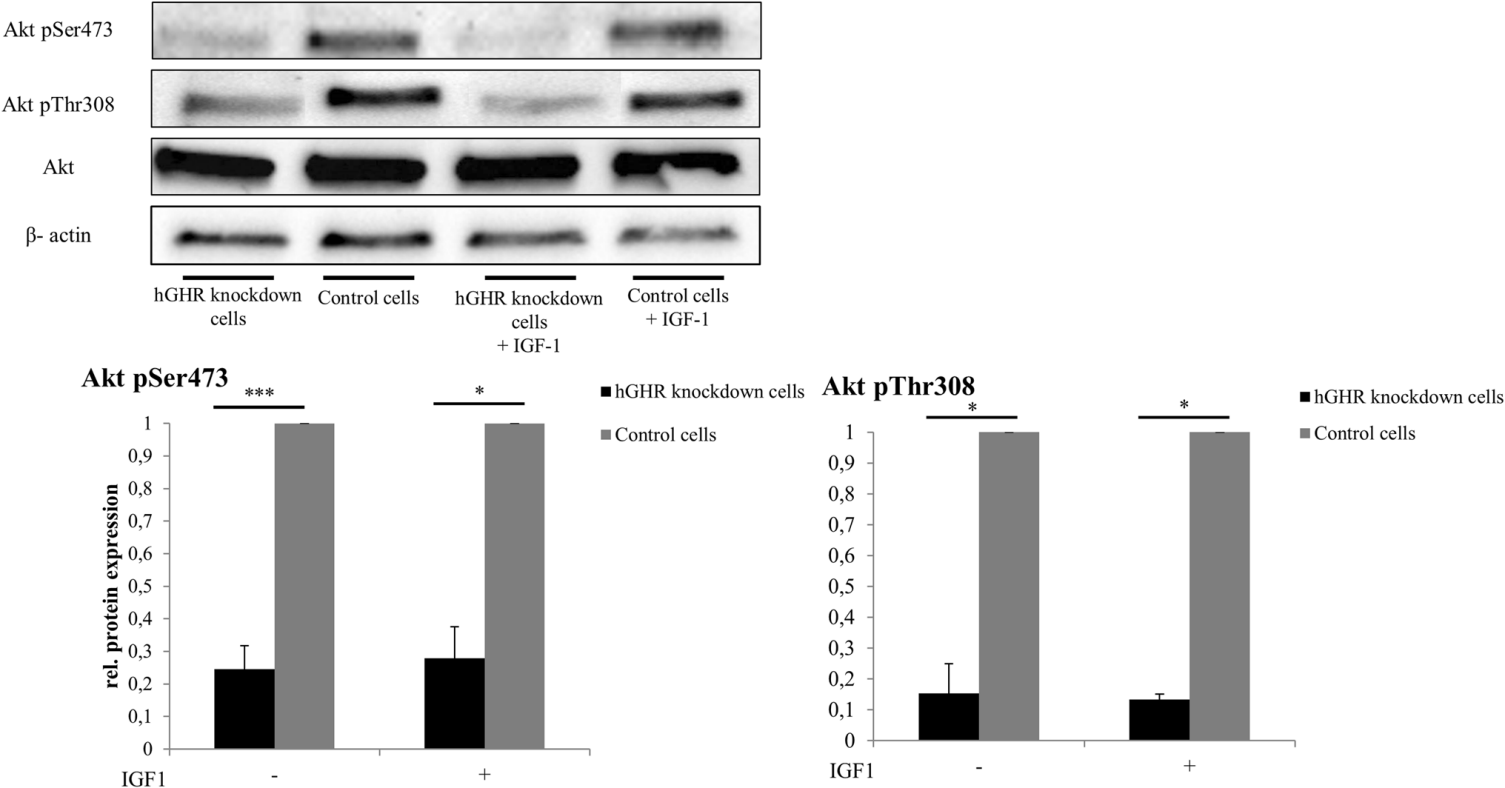
pAKT/AKT in GHR-knockdown cells. Ratio of Threonine308 or Serine473 phosphorylated AKT/AKT (pAKT/AKT) was determined by Western blot analysis. To investigate whether IGF-1 compensates for significantly reduced pAKT/AKT ratio, cells were preincubated with IGF-1 at a concentration of 200 μg/ml

Phosphorylation of Akt (pAKT) was quantified to estimate insulin signaling activity in hepatocytes. As shown in Fig. 4, Threonine308 Akt phosphorylation was significantly decreased by 84 ± 9% (*n* = 4, *p* < 0.001) and Serine473 phosphorylation by 75 ± 7% in GHR-knockdown cells compared to non-silenced control HepG2 cells (*n* = 4, *p* = 0.021). Incubation of GHR-knockdown cells with IGF-1 at a concentration of 200 µg/ml for 24 h did not abolish negative effects on Akt phosphorylation in GHR-suppressed cells.

M-RNA levels of G6Pase were significantly increased by 57 ± 20% in GHR-suppressed cells (*n* = 4; *p* = 0.015). Expression levels were also significantly higher in IGF-1 pre-incubated GHR-suppressed cells (*n* = 4; *p* = 0.039). IGF-1 supplementation led to a significant increase in G6Pase expression in both GHR-knockdown and control cells. M-RNA levels of PC and PCK1 tended to be higher in GHR-knockdown cells than in control cells without reaching statistical significance (PC: + 37.58 ± 13%, *p* = 0.10, PCK1 + 30.40 ± 0.40, *p* = 0.53, *n* = 3).

M-RNA expression levels of DGAT2, PPARγ, PPARα, SREBP1c, CPT1, FAS and SCD-1 were similar in GHR-knockdown and control cells.

## Discussion

Obesity is frequently associated with insulin resistance, glucose intolerance and fatty liver disease. It is also characterized as a state of low GH function resulting from blunted GH secretion or increased GH clearance [14, 15]. Significant weight loss not only improves glycemic control and fatty liver disease [16, 17] but is also accompanied by an amelioration or restoration of GH metabolism [18–20].

Several studies suggested a direct relationship between dysregulated GH metabolism and pathophysiology of NAFLD. In a cross-sectional study, low GH levels were associated with higher NAFLD prevalence [4, 21]. Recently, Collin de l´Hortet et al. [22] described involvement of reduced GH/epidermal growth factor receptor (EGFR) signaling in diminished liver regeneration capacity in hepatic steatosis which was partly resolved by GH administration. Remarkably, increased de novo lipogenesis and fatty liver were observed in male adult onset hepatocyte-specific GHR-knockdown mice [9].

In this study, we aimed to define metabolic consequences of reduced hepatic growth hormone action and characterize its role in NAFLD.

In our cell culture model, GHR knockdown was associated with decreased basal and insulin-stimulated glycogen content suggesting decreased hepatic insulin sensitivity. Mechanistically, pronounced reductions in Akt phosphorylation suggest diminished insulin signaling in GHR-knockdown HepG2 cells. The latter might also explain increased expression levels of G6Pase. While fatty acids are known to elevate G6Pase expression [23], the opposite has been shown for insulin [24]. By catalyzing the terminal step of gluconeogenesis, G6Pase is a critical determinant of hepatic glucose output that is characteristically increased in states of insulin resistance and type 2 diabetes.

Remarkably, administration of IGF-1 did not fully compensate for harmful metabolic effects in GHR knockdown suggesting that GHR effects on hepatic glucose metabolism are mainly independent of IGF-1. We speculate that reduced activation of the mitogen-activated protein kinase (MAPK)/extracellular signal-regulated kinase (ERK) pathway which was found to influence insulin sensitivity by downregulating insulin-like receptor gene expression [25] contributes to decreased insulin signaling in hepatocytes with GHR knockdown. In accordance with our data, Liu and colleagues [9] showed in their liver-specific GHR-deficient mouse model that alterations in hepatic fatty acid and glucose metabolism are not mediated by IGF-1 but result from hepatic GH resistance. In contrast to HepG2 cells, IGF-1R is not expressed in healthy hepatocytes. However, IGF1-R expression was detected in liver samples of patients with liver diseases including hepatitis C virus infection [26].

Addressing the question whether reduced hepatic GH action in NAFLD might contribute to frequently found glucose intolerance, we investigated GH metabolism in liver biopsies of patients at various stages of NAFLD. While hepatic GHR mRNA expression levels were similar, IGF-mRNA expression was significantly reduced in patients with NASH when compared to obese females with simple steatosis. Fasting glucose levels and prevalence of overt type 2 diabetes were also significantly higher in patients with NASH when compared to obese subjects with simple steatosis.

In our study, decreased hepatic IGF-1 mRNA expression was not accompanied by reduced hepatic GHR expression. We hypothesize that diminished IGF-1 mRNA expression might result from reduced GH secretion in these subjects. Increased levels of free fatty acids and hyperinsulinemia have been reported to suppress GH secretion partly by modulating binding proteins of IGF-1 before [15]. Our conclusions are limited by lacking systemic GH metabolism data. Future studies will be necessary to discriminate in more detail hepatic from systemic GH effects on progression of fatty liver disease in humans.

Our results suggest that beyond other mechanisms, decreased hepatic GH action in NASH further deteriorates glucose tolerance by increasing hepatic glucose output. Low GH action in obesity might also lead to metabolic alterations of other tissues such as the adipose tissue and skeletal muscle that might also contribute to deteriorated insulin sensitivity and glucose tolerance in obese patients with NASH.

The latter might result from diminished insulin signaling leading to decreased glycogen storage and enhanced gluconeogenesis. Our data and conclusions are restricted to obese patients with NASH only.

In summary, our studies suggest that reduced hepatic GH action in obese subjects with NASH has detrimental effects on glucose tolerance by diminishing hepatic insulin signaling and increasing hepatic glucose output. Attenuated hepatic GH signaling might link steatohepatitis with insulin resistance in obesity.
